# Supplementary figures and images for: ADAR-mediated regulation of PQM-1 expression in neurons impacts gene expression throughout C. elegans and regulates survival from hypoxia
Source: PLoS Biol. 2023 Sep 25;21(9):e3002150. doi: 10.1371/journal.pbio.3002150 (PMC10553819; doi:10.1371/journal.pbio.3002150)

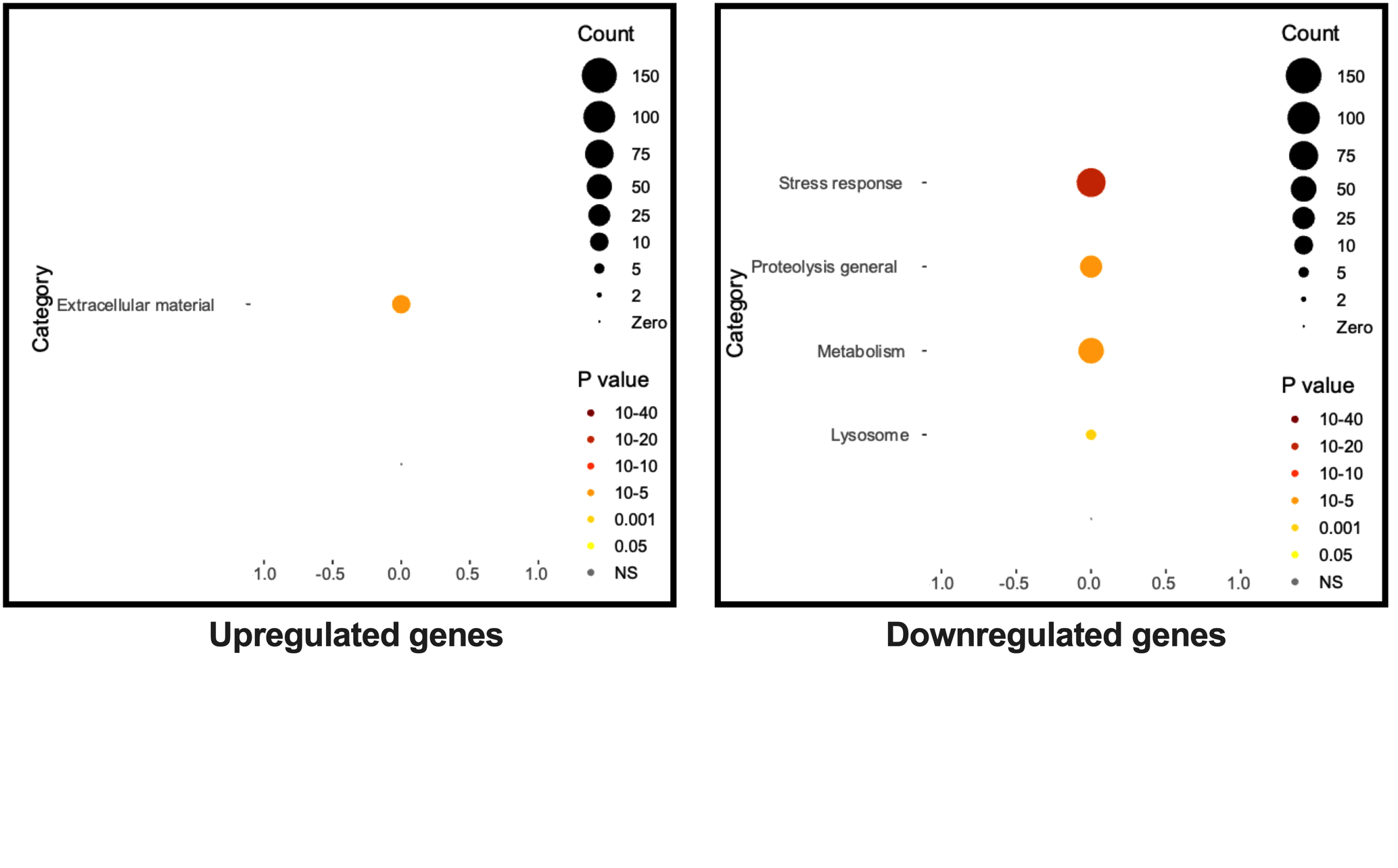

Supplement: S1 Fig — For the total number of input genes in each of the categories (regulated gene set), the P value is calculated using Fisher’s exact test. “Count” indicates the number of genes within a specific category. The size and color of the circles for each of the categories signifies the number of genes (size) and P value (color) for the categories mentioned (see key in figure). (TIFF) [file pbio.3002150.s001.tiff]

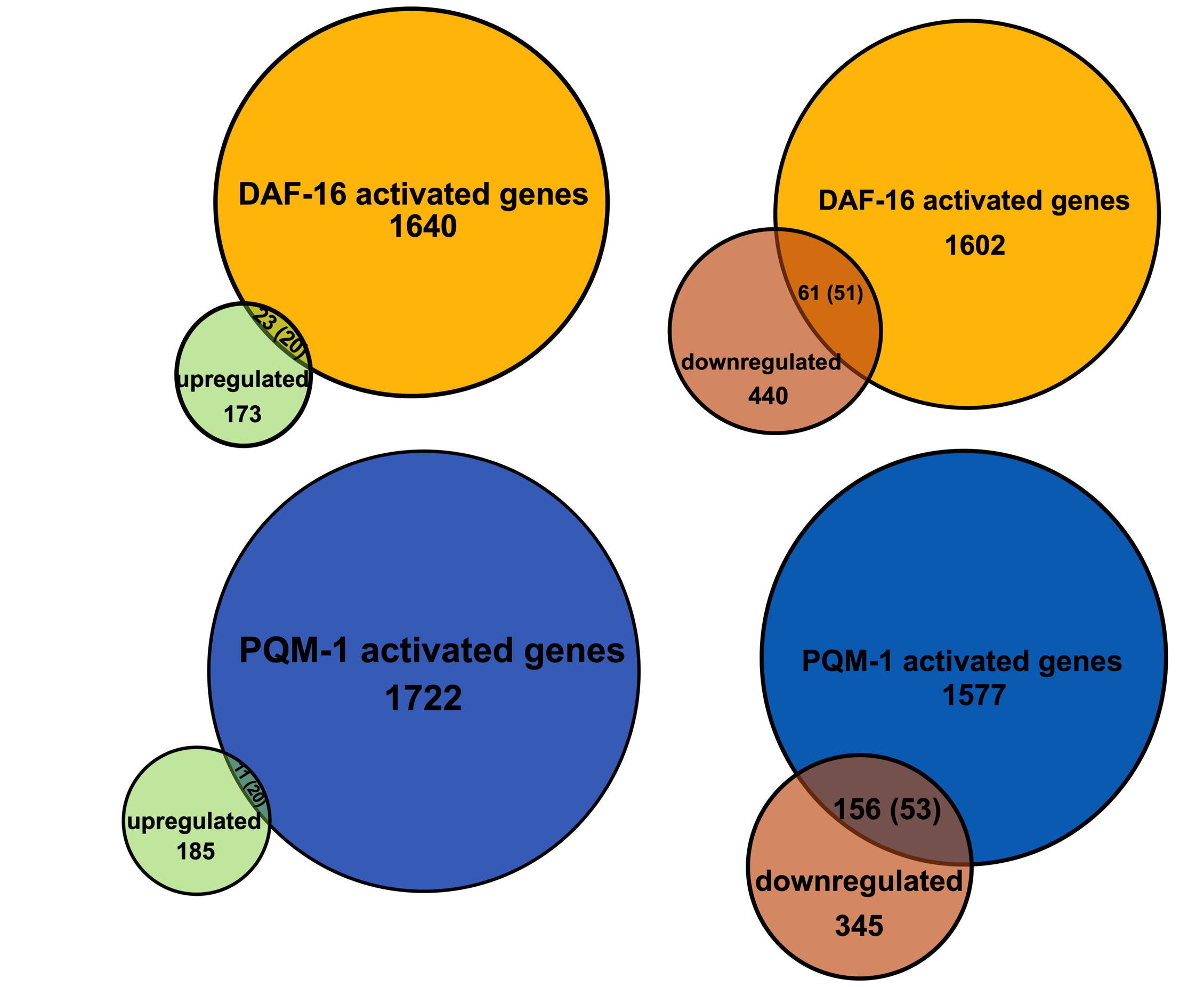

Supplement: S2 Fig — DAF-16 activated (yellow circle) and PQM-1 activated (blue circle) genes from Tepper and colleagues were individually overlapped with the genes found to be up-regulated (green circle) and down-regulated (light red circle) genes in adr-2(-) neural cells compared to wild-type neural cells. The number in parentheses denotes the number of overlapped genes between the 2 datasets expected due to random chance. (TIFF) [file pbio.3002150.s002.tiff]

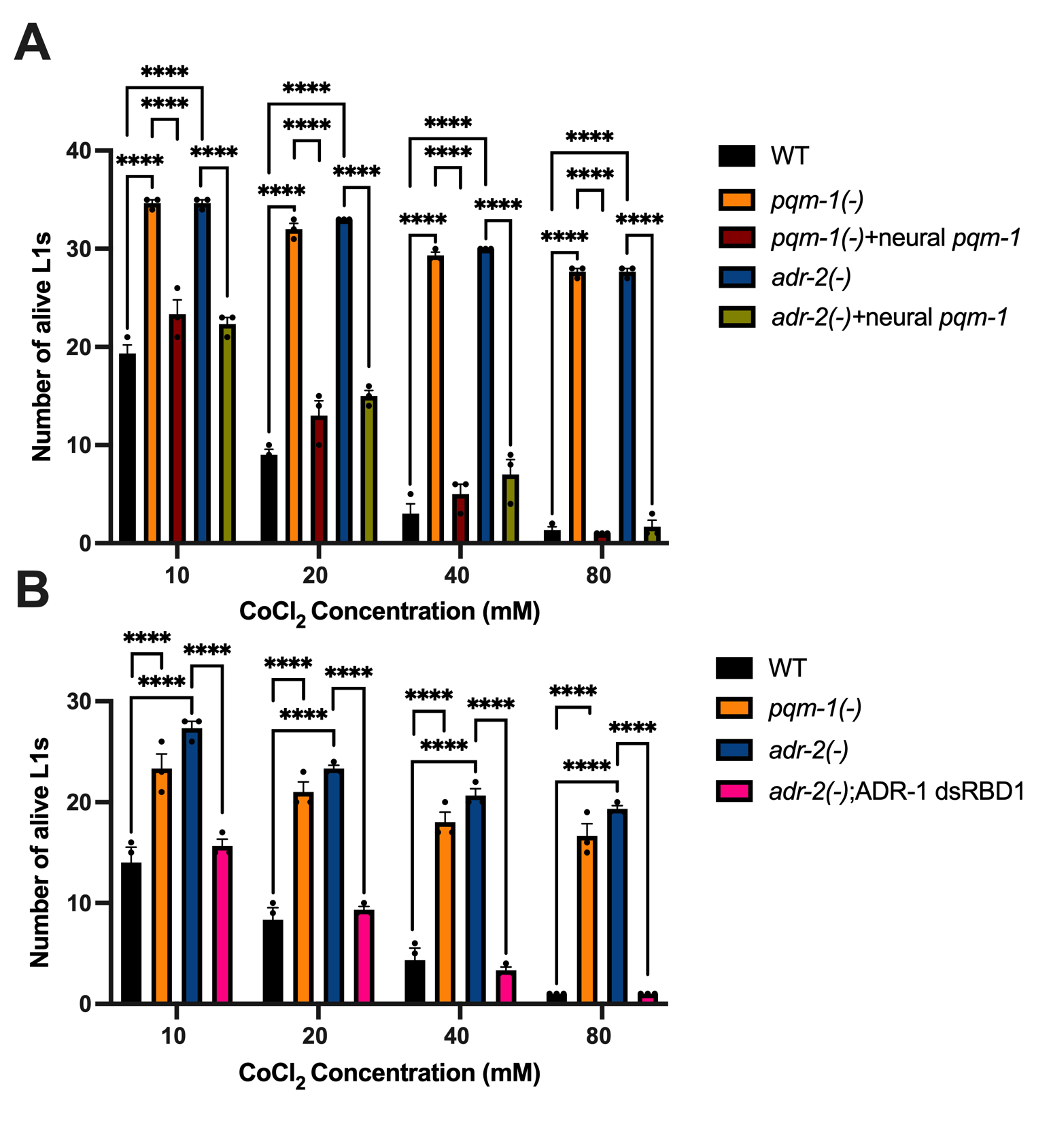

Supplement: S3 Fig — (A, B) Survival of transgenic (A) and non-transgenic (B) hatched L1 animals with number of alive L1s on Y axis and varying concentrations of CoCl2 on X axis. Error bars represent standard error of the mean (SEM). Statistical significance across strains was calculated using two-way ANOVA test. ****p < 0.0001. All individual data and statistics are included in S5 Data under Supporting information. (TIFF) [file pbio.3002150.s003.tiff]

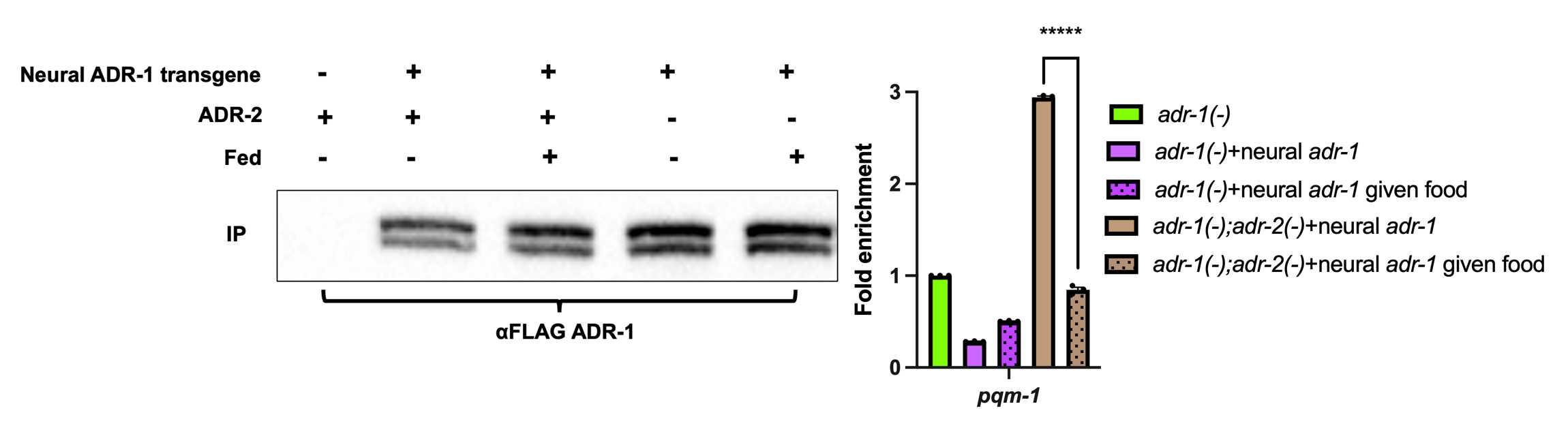

Supplement: S4 Fig — Bar graph represents the fold enrichment determined by dividing the IP/Input value from qPCR for the indicated strains divided by that of negative control. The mean of 3 biological replicates was plotted. Error bars represent SEM. Statistical significance was calculated by multiple unpaired t tests followed by Holm–Šídák multiple comparisons correction. *****p < 0.000001. All individual data and statistics are included in S6 Data under Supporting information. (TIFF) [file pbio.3002150.s004.tiff]

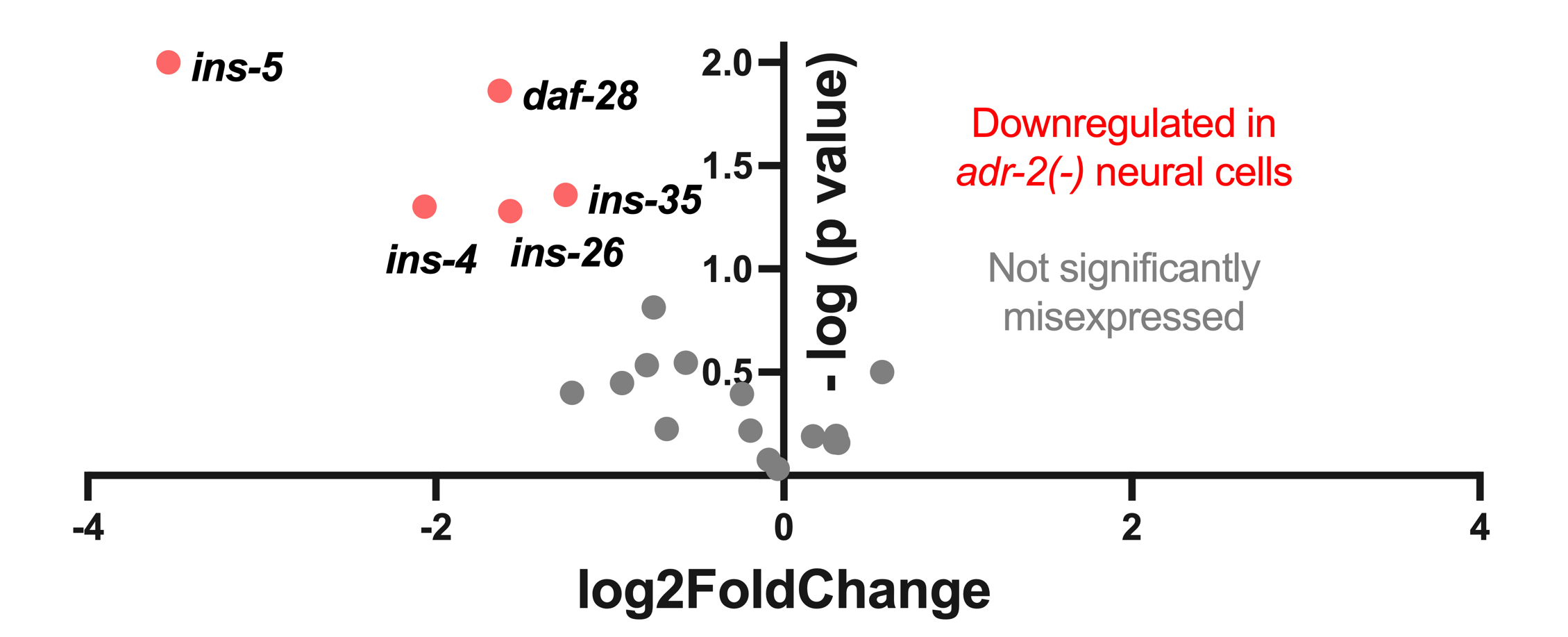

Supplement: S5 Fig — Plot depicting expression of the 20 moderately expressed (read counts between 50 and 300) or highly expressed (read counts >300) ILPs in adr-2(-) neural cells from the neural RNA sequencing dataset. Red dots indicate ligands that have significantly decreased expression in adr-2(-) neural cells compared to wild-type neural cells and are annotated. ILPs with p value <0.05 and log2fold change <-0.5 were considered significantly down-regulated. All individual data and statistics are included in S7 Data under Supporting information. (TIFF) [file pbio.3002150.s005.tiff]
