# Supplementary material for: ADAR-mediated regulation of PQM-1 expression in neurons impacts gene expression throughout C. elegans and regulates survival from hypoxia
Source: PLoS Biol. 2023 Sep 25;21(9):e3002150. doi: 10.1371/journal.pbio.3002150 (PMC10553819; doi:10.1371/journal.pbio.3002150)
Supplement: S1 Raw Images — (PDF) [file pbio.3002150.s015.pdf]

Raw images for Figure 4B

Western Blot images- Images were taken on the ImageLab 6.1 software using the Bio-Rad Chemidoc MP. Imaging was done using Chemi Hi Sensitivity. To obtain the blot on the left with the ladder, a multichannel scan was taken with the bands under the red channel (Chemi Hi Sensitivity setting) and the ladder on the green (colorimetric setting). The blot on the right is after 5 seconds exposure.

Input samples (three biological replicates)

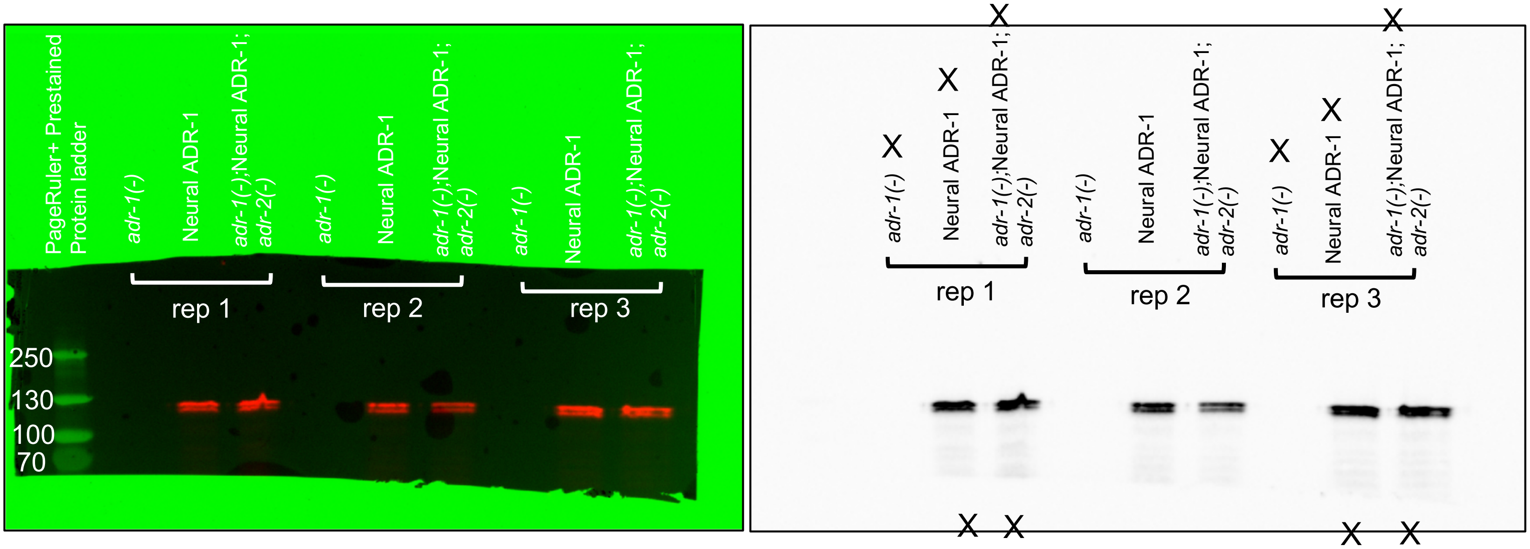

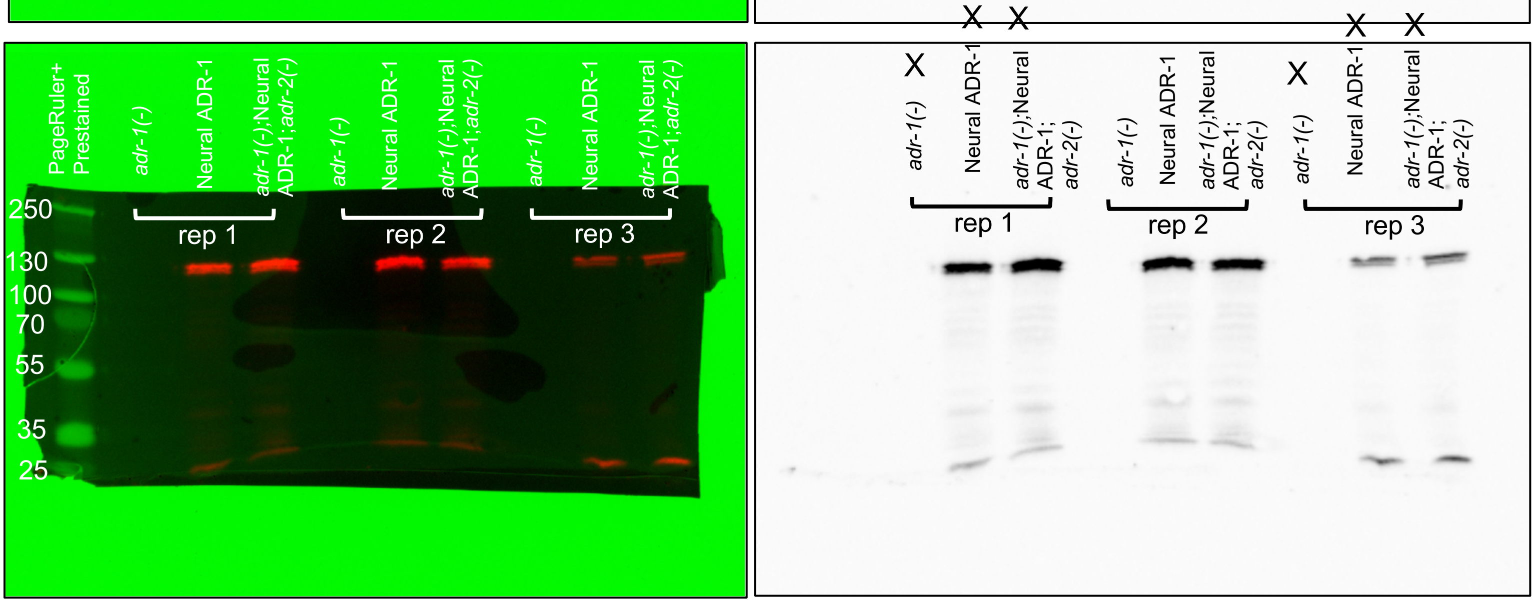

IP samples (three biological replicates)
