# Supplementary material for: ADAR-mediated regulation of PQM-1 expression in neurons impacts gene expression throughout C. elegans and regulates survival from hypoxia
Source: PLoS Biol. 2023 Sep 25;21(9):e3002150. doi: 10.1371/journal.pbio.3002150 (PMC10553819; doi:10.1371/journal.pbio.3002150)
Supplement: S2 Raw Images — (PDF) [file pbio.3002150.s016.pdf]

Raw images for Supplemental Figure S4

Western Blot images- Images were taken on the ImageLab 6.1 software using the Bio-Rad Chemidoc MP. Imaging was done using Chemi Hi Sensitivity. The blots are after 1 second exposure.

	X	X	X	X	X	X	X	X	X	X
Neural ADR-1 transgene	-	+	+	+	+	-	+	+	+	+
ADR-2	+	+	+	-	-	+	+	+	-	-
Fed	-	-	+	-	+	-	-	+	-	+

Rep 1

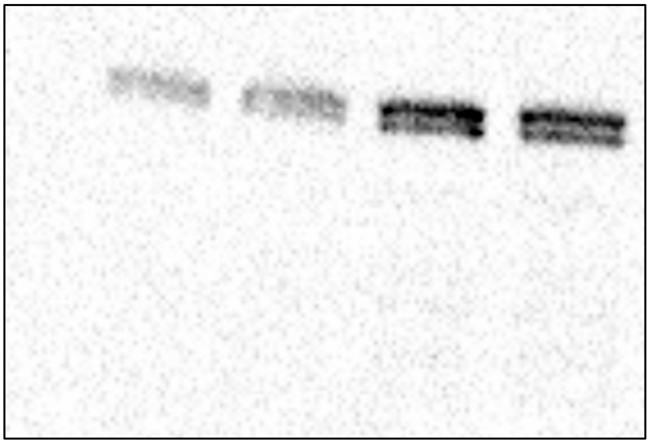

Inputs

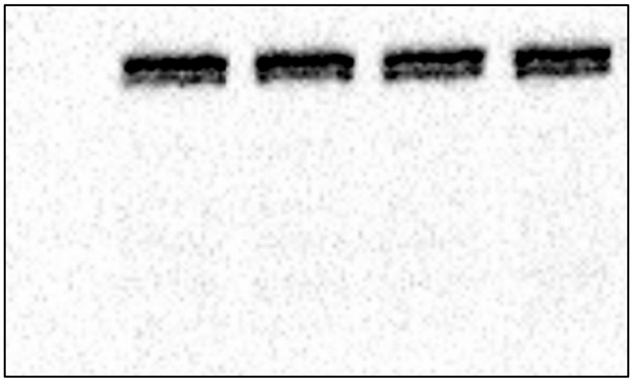

IPs

	X	X	X	X	X					
Neural ADR-1 transgene	-	+	+	+	+	-	+	+	+	+
ADR-2	+	+	+	-	-	+	+	+	-	-
Fed	-	-	+	-	+	-	-	+	-	+

Rep 2

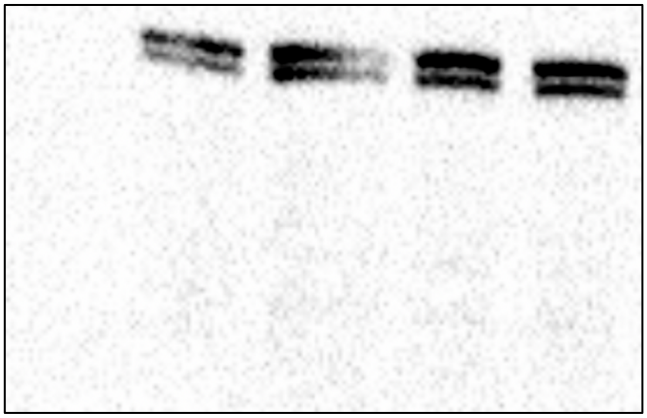

Inputs

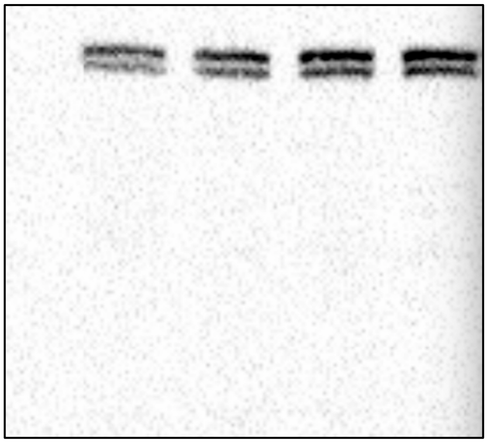

IPs

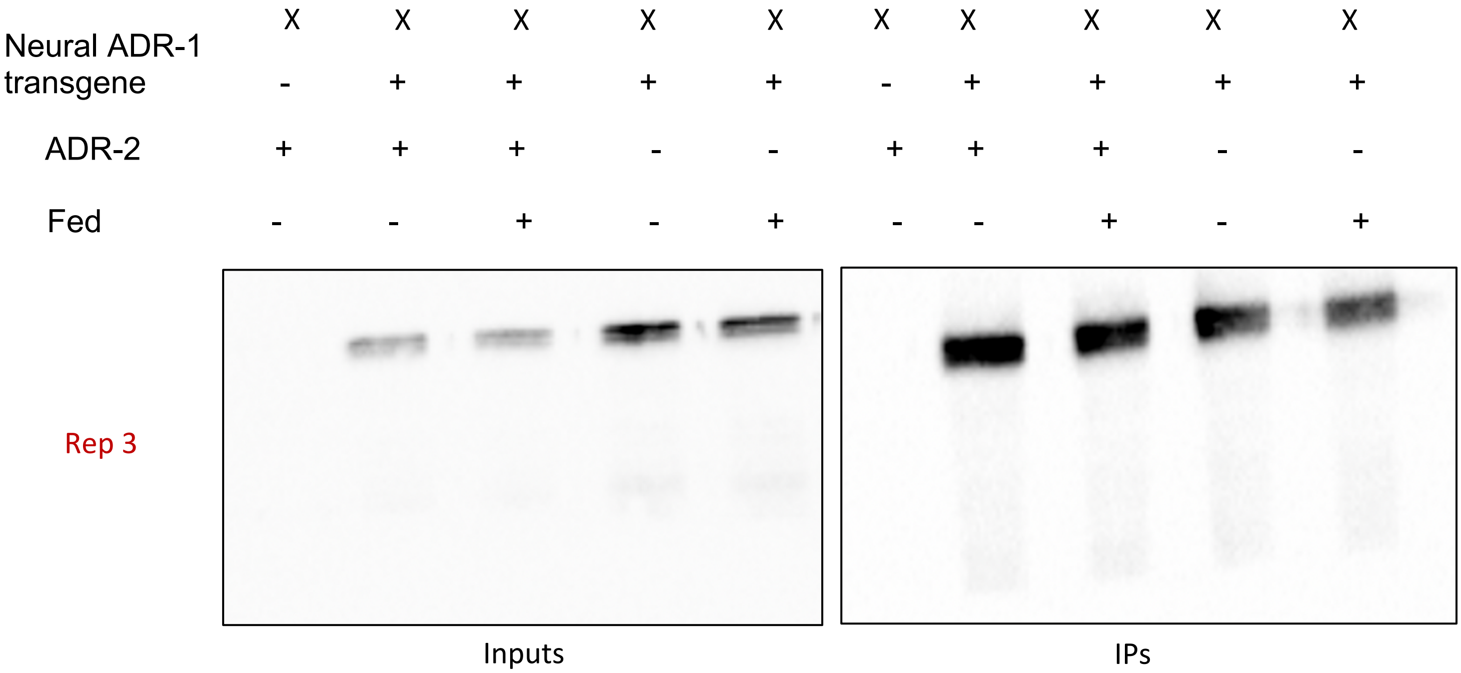
X X X X X X X X X X
Neural ADR-1 transgene
- + + + + - + + + +
ADR-2
+ + + - - + + + - -
Fed
- - + - + - - + - +
Rep 3
Inputs
IPs
