# Supplementary material for: ADAR-mediated regulation of PQM-1 expression in neurons impacts gene expression throughout C. elegans and regulates survival from hypoxia
Source: PLoS Biol. 2023 Sep 25;21(9):e3002150. doi: 10.1371/journal.pbio.3002150 (PMC10553819; doi:10.1371/journal.pbio.3002150)
Supplement: Attachment — Submitted filename: Response to reviewers.pdf [file pbio.3002150.s017.pdf]

Point-by-point response to the reviewer's comments. Changes in the manuscript are in red.

Reviewer #1

We thank the reviewer for noting the thoroughness of the study and the identification of an important role for ADR-1 and ADR-2 in regulating insulin signaling and survival from hypoxia in *C. elegans*.

Major concerns:
1. The authors didn't access pqm-1 RNA expression in the experiments that generated Figure 4A and 4C. Specifically, does pqm-1 RNA expression remain the same as WT when both adr-1 and adr-2 are deleted (as does expression of dod-17, dod-19 and dod-24). Or for panel C, does the loss of ADR-1 dsRNA binding ability prevent reduced expression of pqm-1 RNA in the absence of adr-2. These data would greatly support the rest of the manuscript.

The reviewer brings up excellent points and data needed to extend the ADR-1 results. We do agree that we could check *pqm-1* expression in neural cells from all the mutants mentioned in Figures 4A to confirm the downstream gene changes are due to *pqm-1* levels. However, the neural isolation is costly, and we believe that comparing *pqm-1* expression in neural cells from *adr-2(-)* animals and *adr-2(-)* animals lacking ADR-1 dsRNA binding ability is the strongest piece of evidence to support the main conclusions in the manuscript. To address this concern, we generated GFP expressing lines to sort neural cells from the strains in Figure 4C. Consistent with the overall model, we observed that loss of ADR-1 RNA binding in the *adr-2(-)* animals restores *pqm-1* expression in neural cells. This data has been added as Figure 4D to the manuscript and described in the text on the top of page 9.

2. The authors show that feeding the L1 arrested animals can rescue the phenotype of adr-2 loss. This raises a question of how that influences regulation of pqm-1 RNA by ADR-1. In the discussion the authors suggest that feeding could influence ADR-1 binding to pqm-1 RNA. One possibility is that after feeding another transcription factor (DAF-16) is driving expression of DOD-17 DOD-19 and DOD-24. The authors could assess PQM-1 expression as they did for the other genes in Figure 1C. Is PQM-1 expression rescued by feeding? Or possibly ADR-1 expression is reduced upon feeding? There are several possibilities here that the authors should discuss further (in a separate paragraph) and some experiments that could be done to strengthen the manuscript.

The reviewer brings up great points about feeding rescuing the decreased gene expression of the PQM-1 activated genes observed in the *adr-2(-)* animals. This observation raises the possibility of another transcription factor driving the expression of these genes under fed conditions or that there is no decreased neural *pqm-1* expression upon loss of *adr-2* when the animals are fed. We have included these

possibilities in the discussion section (top of page 11). While the current data does not distinguish between these possibilities, our results also show that neural ADR-1 binding to *pqm-1* causes decreased *pqm-1* expression in *adr-2(-)* neural cells. Therefore, to further dissect the mechanism of feeding affecting *pqm-1* expression, we tested the possibility that ADR-1 binding to *pqm-1* was altered upon feeding. This data has been included as Supplemental Figure S4 in the manuscript and has been discussed on the top of page 11. Briefly, we do observe that as suggested by the reviewer, feeding does influence ADR-1 binding to *pqm*-1 and that ADR-1 no longer binds *pqm-1* in fed L1

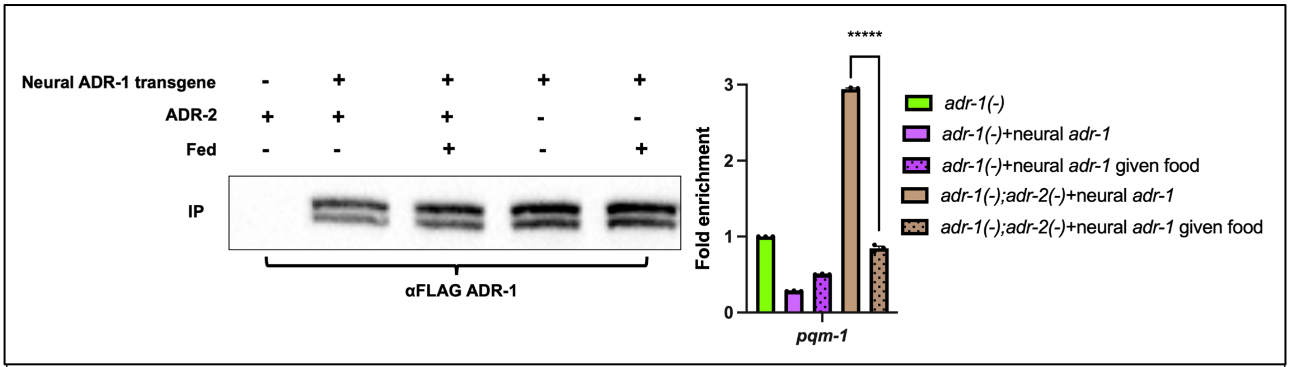

Included as Supplemental Figure S4: Neural ADR-1 binds *pqm-1* in the absence of *adr-2* specifically in starved L1 animals. Bar graph represents the fold enrichment determined by dividing the IP/Input value from qPCR for the indicated strains divided by that of negative control. The mean of three biological replicates was plotted. Error bars represent SEM. Statistical significance was calculated by multiple unpaired t-tests followed by Holm-Šídák Multiple Comparisons Correction. *****$p < 0.000001$.

animals lacking *adr-2* (Supplemental Figure S4). Additionally, we are actively pursuing this question further with a global set of ADAR targets that are influenced by environmental factors via direct ADAR binding and believe that this data is beyond the scope of this manuscript. We also find the influence of factors such as feeding on what substrates ADARs bind extremely fascinating.

Minor concerns:

1. Is it possible that the CoCl2 treatment used as a hypoxia mimic be activating the insulin/daf-2/pqm-1 pathway via another means? Given Co is a heavy metal, it may be activating other stress pathways leading to the observed phenotypes. Is there another way of doing this experiment, or an additional control that could be added to address this concern?

We thank the reviewer for raising these concerns. As cobalt mimics a hypoxic state by rendering EGL-9 inactive (Epstein et al., *Cell*, 2001), to clearly demonstrate that PQM-1 is functioning to negatively regulate survival via the classic HIF pathway and not a general response to heavy metals, we could perform our $CoCl_2$ assays in the *egl-9* mutant background. However, it has also been demonstrated in worms that $CoCl_2$ treatment can result in transcriptional responses that are both HIF-dependent and non-HIF-mediated (Padmanabha et al., *Genetics*, 2015). HIF-independent pathways that function in hypoxia and are still being discovered (ex. Doering et al., *eLife*, 2022) and thus, testing mutants in each of these pathways would be difficult. The most straightforward alternative approach to studying the role of PQM-1 in hypoxia is to perform assays with a hypoxia chamber. In fact, the previous study by *Heimbucher et*

al, 2020 was able to demonstrate a role for PQM-1 in negatively regulating survival to hypoxia using this assay – which involves incubation at low oxygen conditions followed by a normoxic period and monitoring cell survival. We could perform similar analyses with access to a chamber. However, some concerns arise as to prolonging L1 arrest during the normoxic period and whether that in itself would influence *pqm-1* levels. We have included these concerns and limitations on the top of page 13.

2. The authors use a two-way ANOVA for several analysis where this reviewer doesn't think it is the appropriate test. Typically, that test would be used for experiments with two variables that are changing (like genotype (WT vs. Mut.) and concentration of a drug (0,1,10, etc.)). That doesn't seem to be the case in places where it is used. Furthermore, it would most likely need a post-hoc test to identify which comparisons are significant.

We thank the reviewer for pointing out these important points about the statistical tests. To be more thorough to all readers, we have added a paragraph in the methods section detailing the program used for statistical analysis. In addition, we agree for the data comparing only two variables, a more accurate statistical test is multiple t-test. Figures 1C, 1D, 2A, 2C, 3C, 3D, 4A and 4C have been changed according to the correct statistical test (multiple t-tests) and this is reflected in the figure legends. Despite this change in statistical analysis, all conclusions stated as significant in the initial submission remain significant.

3. The authors use 'multiple t-tests' for some comparisons but don't describe which method of multiple comparison correction they are using.

We apologize for omitting this crucial information about the multiple comparison correction. The multiple comparisons correction used for all the panels in Figures 1,2,3 and 4 have now been added to the figure legends.

4. The description for Figure 2B is lacking. It would also be nice to include summary statistics for all of the animals imaged.

We thank the reviewer for bringing this important point to our attention and apologize for the lack of summary statistics in the initial submission. We have added more details to the Figure 2B description and have also included the summary statistics for the imaged animals. The quantification method used has also been added to the "Fluorescence Microscopy" subsection of the methods section.

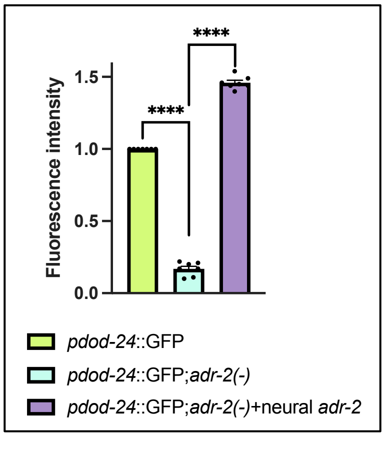

Included in Figure 2B: The bar graphs below the images represent the summary of fluorescence intensity quantification using FIJI software for all the animals imaged. Data from 7 animals is plotted where each dot represents one animal. The values were normalized to *pdod-24*::GFP. Statistical significance was determined using an ordinary one-way ANOVA test. ****p <0.0001

Reviewer #2:

We thank the reviewer for the kind description of our experiments and manuscript and well as appreciated that our study provides insights into how the nervous system adapts to environmental cues and promotes survival in hypoxic conditions through post-transcriptional gene regulation.

However, prior to publication two points should be addressed.
1) Figure 2B shows only one organism in the field. It would be important to provide this micrograph with some statistics on more worms.

We agree with the reviewer and apologize for this oversight. As discussed above in the response to reviewer #1, we have included the summary statistics for the imaged animals under the images as a part of Figure 2B.

2) Can the authors speculate what is happening to pqm-1 RNA in the absence of ADR-1? Does pqm 1 contain a structure element that might serve as a target for a dsRBD? Is translation affected, leading to a destablization of pqm-1? Are there experiments testing for ribosome profiling or RNA stability in the presence or absence of adr-1.?

We appreciate the reviewer's interest in delving further into the mechanism of how ADR-1 binding affects *pqm-1* expression and have added some text to the discussion of the manuscript on the bottom of page 10/top of page 11. As to question of what molecular process ADR-1 is impacting, we think that it is likely to be RNA stability and not translation as we see impacts on mRNA levels. Furthermore, there is precedence for ADARs to bind and regulate mRNA stability. A great way to examine this would be to test *pqm-1* stability in the presence and absence of *adr-1* using transcriptional inhibitors such as Actinomycin D. However, the data in the manuscript demonstrates that *pqm-1* is differentially expressed specifically in the nervous system. We believe that it would be technically challenging to isolate neural cells from L1-arrested animals with and without Actinomycin D exposure over a time course to monitor *pqm-1* stability.

At this point, it is possible that ADR-1 could either be affecting *pqm-1* stability by blocking the binding of stabilizing factors or facilitating the binding of certain destabilizing factors (either proteins or small RNAs) to the *pqm-1* transcript. We think the best way to get at the factors and mechanism of downregulation would be to perform an unbiased genetic screen and plan to pursue this in the future.

Does pqm 1 contain a structure element that might serve as a target for a dsRBD?

This is an important question, and we appreciate the reviewer raising it. We are actively thinking about this question both in the broad sense and for *pqm-1*. Currently, there is no consensus in the field for dsRNA binding proteins to recognize a specific structure *in vivo*. Ongoing work in the Hundley lab is focused on this question and is performing unbiased transcriptome-wide analysis of RNA binding by *C. elegans* ADR-1 and ADR-2. Interestingly, our current study suggests we also need to do that analysis in different

genetic backgrounds! Early structural work on dsRNA binding proteins does indicate about 20 basepairs of dsRNA are needed for *in vitro* binding and using a structure prediction program – mFold – we have obtained the predicted RNA structure of the entire *pqm-1* transcript (Response Figure 1A). Since ADARs can bind intronic regions, we used the unspliced sequence of *pqm-1* to obtain a structure prediction. While there are clearly double-stranded regions within the transcript where ADR-1 could potentially bind, there is no striking region to delete or mutate. To be more comprehensive, the predicted RNA structure for the *pqm-1* spliced sequence was also obtained (Response Figure 1B), however no striking dsRNA region was observed. Since ADARs are known

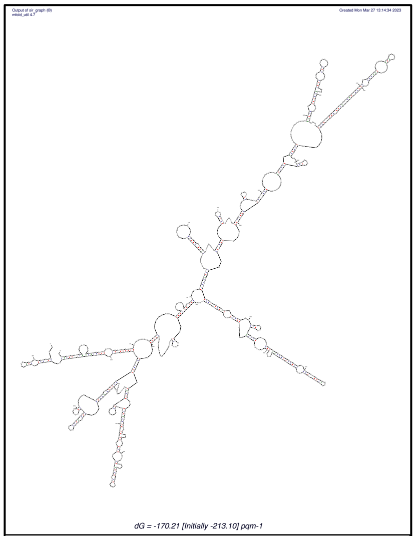

Response Figure 1A: Predicted *pqm-1* RNA fold structure from inputting the *pqm-1* unspliced sequence on the http://www.unafold.org/mfold.php website.

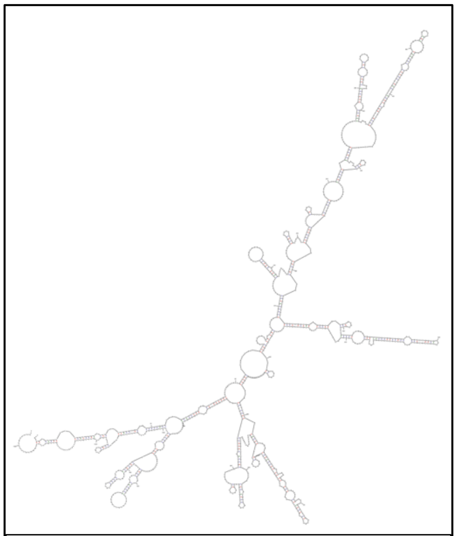

Response Figure 1B: Predicted *pqm-1* RNA fold structure from inputting the *pqm-1* spliced sequence on the http://www.unafold.org/mfold.php website.

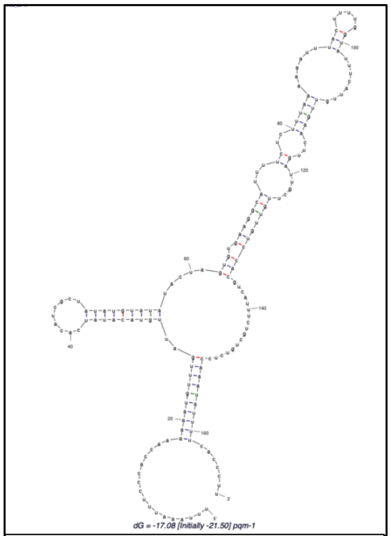

Response Figure 1C: Predicted *pqm-1* RNA fold structure from inputting the *pqm-1* 3' UTR sequence on the http://www.unafold.org/mfold.php website.

to bind 3'UTR regions of transcripts, we obtained the RNA structure of the *pqm-1* 3' UTR region using the same software. Similar to the unspliced and spliced *pqm-1* sequences, no striking dsRNA regions were observed in the *pqm-1* 3' UTR region. (Response Figure 1C). We would like to create *pqm-1* reporters lacking individual regions and assess ADR-1 binding. However, we feel this is outside the scope of the current manuscript.

Reviewer #3:

1. The authors show RNA seq data from purified neurons. While they cite this RNA seq from a separate study, it would be important to show controls for the specificity of the purification.

We agree with the reviewer that specificity of purification should be demonstrated for this technique and apologize for not making this more clear. This technique was established in our laboratory in a manuscript published in *eLife* in 2017 (*Deffit et al, 2017*). In that study, we have discussed the neural cell isolation in great detail and

monitored the expression of both neural and non-neural genes to validate the purity of the isolated neural cell samples. For the data presented in Figure 1A, the isolation as well as qPCR for neural and nonneural genes was published in our 2021 *Genome Research* manuscript (*Rajendren et al, 2021*) in Supplemental Figure S1.

For the data presented in Figure 1B and 3A, we used neural cells isolated by the authors of the 2021 *Genome Research* manuscript. For the data presented in new Figure 4D, we performed neural cell isolation and validated purity using the same published method and the data on purity of the isolation is shown below.

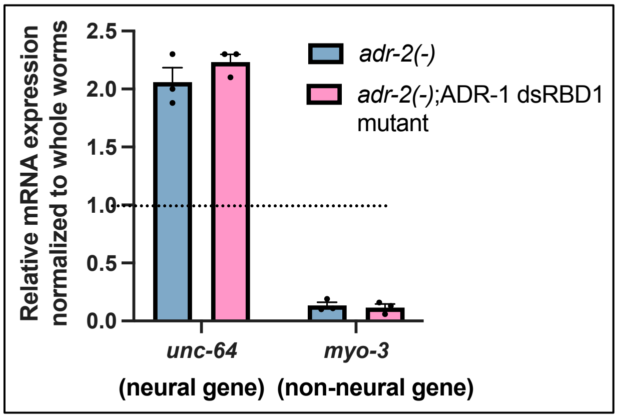

Response Figure 2: Validating purity of isolated neural cells in Figure 4D. Expression of neural gene *unc-64* and muscle gene *myo-3* was determined relative to housekeeping gene *gpd-3*. mRNA expression in control whole worms was normalized and set to 1 (dotted line) and relative expression of neural mRNA was plotted with SEM.

More importantly, how do these gene compare with daf-2 regulated genes, or genes regulated by other lifespan-regulating pathways such as the TGF-B pathway, mitochondrial mutants or the folate cycle?
This type of analysis would be essential for linking ard-2 and insulin signaling. It is not sufficient to say that sharing life-span extension shows a connection. WormCat shows only very general categories, are there more specific categories that are also enriched?

We thank the reviewer for raising these concerns and apologize for not making this clear in the manuscript. We also completely agree that simply sharing a common phenotype is not sufficient to say that two genes are connected. We have discussed and performed overlaps between the genes differentially expressed in our dataset and a previously published dataset of all the *daf-2* regulated genes (DAF-16 activated genes and PQM-1 activated genes individually) and this is mentioned in Supplemental Figure S2 of the manuscript. Furthermore, we provide data in the manuscript using *daf-2* and *adr-2*;*daf-2* genetic mutants that support our conclusions about regulation of the genes through the insulin signaling pathway (Figure 1D). Additionally, we also show regulation of the insulin signaling transcription factor (PQM-1) by ADARs (Figures 3A) as well as gene expression profiles of PQM-1 activated genes in rescue experiments using *pqm-1(-)* as well as *adr-2(-)* animals expressing neural *pqm-1* (Figure 3D). Conclusions in the latter half of the manuscript also demonstrate that loss of *adr-2* does not affect insulin signaling globally but affects a specific class of genes that are negatively affected by reduced insulin signaling.

2. The rationale for using the three dod genes appears weak. These genes appear to be decreased in both pure neuronal and entire animal qPCR (Fig1 B and C), therefore these effects appear to be occurring generally. Are these genes the most highly

regulated, or were they cherry picked because they are co-regulated? The conclusion for Fig 1D is that the "expression profile is similar", however, the data simply shows that three genes require either ard-2 or daf-2 for expression.

We thank the reviewer for their concerns. Extensive overlaps were performed between differentially expressed genes in *adr-2(-)* neural cells and *daf-2* regulated genes in Supplemental Figure S2 of the manuscript. According to these overlaps, there were 156 PQM-1 activated genes that were downregulated in *adr-2(-)* neural cells. Since the connection between ADARs and insulin signaling was novel, out of these 156 genes, even though the three "*dod*" or "Downstream of DAF-16" genes were not the most highly regulated, we decided to further pursue these genes since especially by the virtue of their name, these genes are very well established to be regulated by insulin signaling.

3. The data in Figure 4A is insufficient to support one of the major conclusions. The authors use qPCR to compare levels of the three dod genes in ard-1 and -2 mutants with those lacking both genes. The authors conclude that ard-1 has unique functions, as the qPCR values were significant as opposed to adr-2. However, the ard-2 values had a very high standard deviation, thus, the lack of a significance is highly likely to be technical and not biological.

We thank the reviewer for their comments. The conclusion about ADR-1 having unique functions was based on the comparison between *adr-2(-)* and the *adr-1(-);adr-2(-)* animals. As there is no significant difference between wildtype animals and *adr-1(-)* animals but there is a significant difference between *adr-2(-)* and the *adr-1(-);adr-2(-)* animals, this suggests that there is a unique function of ADR-1 specifically in the absence of *adr-2.* We apologize for not making this clearer in the manuscript and have made some changes in the text (page 8) to improve clarity on this important point.

4. Figure 4B shows an RNA precipitation comparing binding of a flag tagged ard-2 transgene. There are several points which make interpretation of this data difficult. First, it is normalized to total RNA and completed in whole animal lysates, therefore neuronal specific effects would be difficult to separate. The normalization to the RNA values in the qPCR appears to provide the baseline in the quantitation (since no protein comes down). This seems difficult to call a baseline value for protein measurement solely on RNA levels, unless the description of the method is unclear. From the blot, it appears the IP'd amounts are quite similar and a lower input amount is what provides the increase in the quantitation. However, with the dynamics of RNA protein interactions, shouldn't the input values be the same, then the differences measured?

We thank the reviewer for their comments and apologize for the lack of clarity. The RNA immunoprecipitation (RIP) assay is a routinely used assay in our lab (*Washburn et al, Cell Reports, 2014, Rajendren et al, NAR, 2018, Ganem et al, Cell Reports, 2019, Rajendren et al, Genome Research, 2021, Oakes et al., JBC, 2017, Raghava Kurup et al, JBC, 2022*). We have added additional details to page 8 and we have added a

reference to the methods to include a book chapter that we wrote that extensively describes the RIP method to improve clarity. In addition, we have modified the figure legend to improve clarity for those less familiar with the methodology.

The experiment in this manuscript uses strains that express FLAG-tagged ADR-1 only in the nervous system, thus IP'd RNA is coming from ADR-1 targets in the nervous system. We have previously used a similar approach to successfully monitor ADR-2 binding specifically in the nervous system (*Rajendren et al, Genome Research, 2021*).

5. The normalization of Figure 4C is done differently than that other assays. Samples are normalized to the mutant, rather than the control. While the authors conclude that a significant increase occurred in the ard-2(-); ARD-1 dsRBD mutant, however, there is no ard-1; ard-2 shown for direct comparison and if the data were normalized to the control, the graph would appear to show that the dsRBD1 mutant simply returns to wild type levels.

We apologize for the normalization change in the initial graph. For this data, we were focused on the differences between the *adr-2(-)* and the *adr-2(-)*;ADR-1 dsRBD1 mutants and thus normalized to *adr-2(-)*. However, to be consistent and improve clarity for all readers, we have re-plotted and normalized the values to the wildtype control. Importantly, none of the conclusions changed. The reviewer is correct that the gene expression in the *adr-2(-)*;ADR-1 dsRBD1 mutants is similar to wildtype levels, which is important. However, the major conclusion is that the presence of the ADR-1 dsRBD1 mutant in the *adr-2(-)* animals results in significantly increased gene expression compared to the *adr-2(-)* animals, which is indicated on the graph.

6. The conclusions from Figure 5 appear inconsistent with the data. In both figures, loss of pqm-1 or adr-2 confer a survival advantage. Expression of neural pqm-1 is insufficient to provide this advantage. To make the conclusion that this effect occurs in the neurons, you would need to show neuronal only expression conferred a different response than loss of expression. In B, there is no adr-2; adr-1 without the RBD as a control. Furthermore, overexpressing transcription factors is prone to artifacts, as they can bind lower affinity sequences.

We appreciate the reviewer's comment and understand the negative regulation can be confusing. We also completely agree that to make the conclusion that the effect occurs in neurons we need to demonstrate a difference between the *pqm-1(-)* animals and the *pqm-1(-)* + neural *pqm-1* animals – which is the exciting part of the data as there is a difference! The *pqm-1(-)* animals survive hypoxia while the *pqm-1(-)* + neural *pqm-1* animals die similar to wildtype animals, suggesting that PQM-1 function solely in neurons is enough to perform its wildtype function. Additionally, since loss of *adr-2* causes decreased neural *pqm-1* expression as shown in the manuscript, we would expect *adr-2(-)* animals to have a similar hypoxic survival as *pqm-1(-)* animals if neural *pqm-1* was a contributing factor in regulating this phenotype - which we also observe!
