# Supplementary material for: ADAR-mediated regulation of PQM-1 expression in neurons impacts gene expression throughout C. elegans and regulates survival from hypoxia
Source: PLoS Biol. 2023 Sep 25;21(9):e3002150. doi: 10.1371/journal.pbio.3002150 (PMC10553819; doi:10.1371/journal.pbio.3002150)
Supplement: Attachment — Submitted filename: Response to Reviewers revision.docx [file pbio.3002150.s018.docx]

*Reviewer #1: I appreciate the authors efforts to address my concerns. The additional experiment showing binding of ADR-1 binding to pqm-1 is influenced by starving is very interesting. I look forward to seeing future work exploring how starvation influences ADR-1 binding.*

We thank the reviewer for the note of excitement in regard to the newly added

data and our future studies.

*Reviewer #2: In this revised version of their manuscript, the authors address all comments raised by me. In particular, for figure 2, a statistics on the GFP fluorescence of pdod::GFP is presented. Further, while the mechanistic consequences of ADR-1 binding to pqm-1 RNA are still not understood, the authors provide a lengthy discussion on the potential mechanisms and also provide related background information that was missing in the original version of the manuscript.*

We thank the reviewer for helping us to strengthen our initial manuscript.

*Reviewer #3: Mahapatra et al present a revised manuscript exploring links between neuronal expression of RNA editing enzymes, expression of a stress responsive gene and treatment with cobalt chloride.  In my initial review, I had concerns about overly broad interpretations of what the assays used in this study can conclude.  In most cases, the authors have not provided sufficient rationale.  The dod genes and pqm genes are being used as sensors.  As their biological functions are not clear, it is important that the specificity of the regulation is rigorously shown, if not the premise of the study is flawed.*

*1.      The authors use several lines of evidence to implicate insulin signaling, however, insulin signaling was not shown to be either necessary or sufficient to the effects in the study.*

*a.      Regulation of Stress Response and Metabolism genes in the adar RNA seq promotes and argument that it is similar to insulin signaling.  Many different biological pathways in C. elegans have these characteristics.  To show there is a specific link, there should a correlation between the genes expressed in both pathways and other data to show specificity.  It is not uncommon to get overlap in RNA seq sets.  There is nothing in this study to show that insulin signaling is the most similar in gene expression.*

We appreciate the reviewer’s concern and agree that a gene ontology of stress response and metabolism is not enough to definitively link insulin signaling to ADARs. We have added a note in the results section (page 4) to indicate these broad categories of gene sets can be influenced by many factors.

b.      Dod genes:  I was concerned that the dod genes were cherry picked from the original data set and were not specific to insulin signaling.  The author's reply was the genes were indeed selected from a based on the name (downstream of daf-16), not expression strength or pathway enrichment.  Although these genes were names based on responsiveness to daf-16, they are regulated in response to around 300 different biological responses listed in the WormBase expression database, therefore there is no basis for using them as a sensor or proxy for insulin signaling.  For example, dod-17 can be regulated by nhr-49 and the PMK-1 map kinase pathway.  These genes are down regulated in daf-2 animals in the present study, but removing daf-2 does not change the adr-2 effects, so it is not possible to make a mechanistic conclusions.

We understand the reviewer’s concerns about the three specific reporter genes

analyzed and have indicated in S2 Fig that there are 153 additional genes

that follow a similar trend (to these three genes) in their regulation by *adr-2* and *pqm-1*.

The observation that gene expression was similar between *adr-2(-)*, *daf-2(-)*

and *adr-2(-);daf-2(-)* animals is a classic genetic epistasis experiment and demonstrates that ADR-2 and DAF-2 function in the same genetic pathway to influence expression of the *dod* genes. To improve the clarity of all readers, we have added this information to the results section on the top of page 5. (Together, these experiments indicate that lack of *adr-2* leads to decreased expression of insulin signaling regulated genes and that both ADR-2 and DAF-2 function in the same pathway to regulate expression of the *dod* genes.)

*2.      I had commented in Figure 4 A that it is not accurate to say that there is no effect with ard-1 based on the lack of statistical significance.  The data points show wide variability; therefore this appears to be an issue of lack of technical reproducibility, not biological variation. This is not sufficiently addressed.*

We understand the reviewer’s concerns and have included three additional biological replicates for Figure 4A. While one of the original biological replicates was a bit of an outlier as mentioned by the reviewer, the data within the other five replicates is very consistent. More importantly, the main conclusions of the figure remain unchanged after inclusion of the additional replicates.

*3.      My concern with the conclusions in the 4B was that the method states the qPCR is normalized to the negative control.  Since there is nothing IP'd in the negative control, what is source of the RNA?*

We thank the reviewer for their comments and apologize for the lack of clarity.

We have amended both the results section on page 8 and the legend to Figure 4 to address this concern. In brief, the beads used for the pulldown can nonspecifically bind RNA molecules; thus, to accurately identify bound RNA binding protein targets, we determine the amount of cDNA present in the negative control samples. However, we do not simply divide by the IP values. For each strain, the cDNA present in the IP values is divided by the cDNA present in the input lysate.

*4.      Regarding figure 5, building mechanistic models based on negative data is problematic, as it is difficult to test alternative hypotheses to establish sufficient rigor.  Figure 5 shows that deletion of pqm-1 has no effect on survival in heavy metal stress.  Expression of neural pqm-1 does limit survival, but overexpression can have many unintended effects.  As I stated in the previous review, B lacks a critical control, the adr-1 without the mutations in the RNA binding domain.*

We agree with the reviewer that building mechanistic models on negative data is problematic. However, we think that the reviewer is misunderstanding the phrase

“negative regulation” and we strongly disagree that deletion of *pqm-1* has “no

effect on survival”. As shown across six individual biological replicates (Figure 5A

and B) and quantified in the supporting information (S3 Fig), wildtype animals die

when exposed to cobalt chloride and animals that lack *pqm-1* have significantly increased survival; this indicates that PQM-1 functions as a negative regulator of

survival.

The intent of the experiments in Figure 5B is to specifically test whether the lack of ADR-1 RNA binding is driving the phenotype in *adr-2(-)* animals. These

experiments are similar to those shown in Figure 4C and 4E. All three of these

experiments include a strain that has ADR-1 without mutations in the RNA

binding domain (the *adr-2(-)* strain has wildtype ADR-1). In addition, Figure 5B

and 4C also have wildtype animals in the same experimental analysis.

*5.      I also agree with the other reviewer that calling the CoCl2 hypoxic stress is overly broad, as it is a heavy metal and induces heavy metal stress.  Although the authors state there are overlaps of this stress with hypoxia, there are independent effects as well. Therefore using hypoxia in the title and throughout the text is not appropriate.*

We are glad that the reviewers are in agreement and the modified text in the initial revision includes the concerns and limitations of cobalt chloride (page 13).
